# A Study of Tapping by the Unaffected Finger of Patients Presenting with Central and Peripheral Nerve Damage

**DOI:** 10.3389/fnhum.2015.00260

**Published:** 2015-05-13

**Authors:** Lingli Zhang, Xiuying Han, Peihong Li, Yang Liu, Yulian Zhu, Jun Zou, Zhusheng Yu

**Affiliations:** ^1^Shanghai University of Sport, Shanghai, China; ^2^Shandong University of Finance and Economics, Jinan, China; ^3^School of Physical Education and Coaching, Shanghai University of Sport, Shanghai, China; ^4^Shanghai Fudan University Affiliated Huashan Hospital, Shanghai, China

**Keywords:** central nerve, peripheral nerve, unaffected hand, dominant hand, finger tapping

## Abstract

**Aim:**

Whether the unaffected function of the hand of patients presenting with nerve injury is affected remains inconclusive. We aimed to evaluate whether there are differences in finger tapping following central or peripheral nerve injury compared with the unaffected hand and the ipsilateral hand of a healthy subject.

**Methods:**

Thirty right brain stroke patients with hemiplegia, 30 left arm peripheral nerve injury cases, and 60 healthy people were selected. We tested finger tapping of the right hands, and each subject performed the test twice.

**Results:**

Finger tapping following peripheral nerve injury as compared with the unaffected hand and the dominant hand of a healthy person was markedly higher than was found for central nerve injury (*P* < 0.05). Finger tapping of the male peripheral group’s unaffected hand and the control group’s dominant hand was significantly higher than the central group (*P* < 0.001). However, finger tapping of the female control group’s dominant hand was significantly higher than the central group’s unaffected hand (*P* < 0.01, *P* = 0.002), the peripheral group’s unaffected hand (*P* < 0.05, *P* = 0.034).

**Conclusion:**

The unaffected function of the hand of patients with central and peripheral nerve injury was different as compared with the ipsilateral hand of healthy individuals. The rehabilitation therapist should intensify the practice of normal upper limb fine activities and coordination of the patient.

## Introduction

Fine movements of hands include percussion, swinging motions, pinching, grasping, pulling, and pushing, among other dexterous functions. Finger tapping has the advantage of being a relatively simple and robust neurologically driven motor task because the inertial and intersegmental interactions are so small that biomechanical influences on movement are reduced (Liu et al., 2006). Measurement of an individual’s ability to tap fingers is an important method of assessing neuromuscular integrity (Collyer et al., 1994). For example, it has been previously shown for handedness (Peters, 1980), for an individual differences in skill acquisition (Ackerman and Cianciolo, 2000), on neurobehavioral effects of toxic agents (Baker et al., 1985), and in clinical neurological examinations (Shimoyama et al., 1990).

Stroke is the third leading cause of death in the world, and it is the leading cause of severe disability in patients in the developed world (Beers et al., 2000). Stroke also adversely affects the quality of life of surviving patients. Nearly 75% of complete stroke survivors are disabled in China every year. Since the unaffected hand of the patient plays an important role in their daily life, whether the function of the unaffected hand would be affected is directly related to the viability and quality of life of the patient.

Upper limb peripheral nerve injury is a common disease in the hand surgery department, most of which are caused by productive labor accidents, such as the cutting, inserting, contusion, or embedding of falling sharp instruments and devices. There are also other reasons why a patient is likely to suffer damage or compressive degeneration along with fracture while daily quality of life and life activities are disrupted, including obstetric brachial plexus palsy (OBPP), and medical accidents following intravenous injections, among other causes. However, such events do not directly threaten one’s life, though such events might lead to motor and sensory neuronal dysfunction of the upper limb, especially with respect to the digits, and even functional losses in the most severe cases. The upper limb peripheral nerve is derived from the cervical and thoracic segment cross-section, and then forms the cervical and brachial plexus. Once a unilateral upper limb peripheral nerve is injured, the dominated organs will be dysfunctional or might suffer complete loss of function. The affected finger sensation and motor function will be decreased to various degrees, and the motor functions of the unaffected side may be correspondingly affected as well.

Whether the unaffected function of the hands of a patient presenting with nerve injury is affected remains clinically inconclusive, and there is a lack of accurate data to demonstrate this empirically. In this study, we used a special instrument to quantify the finger-tapping frequency of the patient, and for convenience, we have abbreviated the finger-tapping frequency as “finger tapping.” We know of many factors that affect finger tapping such as gender, age, hand dominance, neural control, and other factors. Previous research has shown that index finger-tapping correlated significantly with Lind-mark score and was shown to be effective in evaluating hand function in stroke patients (Zhang et al., 2014). In addition, the finger-tapping test has been presented as an embedded measure of performance validity in most standard neuropsychological evaluations (Axelrod et al., 2014). The computerized finger-tapping test is an efficient and precise measure of tapping speed and the kinetics of potential utility in research and clinical studies of motor performance (Hubel et al., 2013). Thus, this task may be used clinically to detect changes of the hemiplegic upper limb during rehabilitation therapy and subsequent recovery.

In the present study, we aimed to understand whether there exists a difference among the finger tapping of central nerve injury to the unaffected hand of the patient, the peripheral nerve injury of the unaffected hand of the patient, and the ipsilateral hand of control healthy control subjects. In the second part of this study, the influence of gender and age on the subjects’ finger tapping was further considered.

## Materials and Methods

### Participants

#### Patients with Central Nerve Injury

We selected 30 patients presenting with stroke of the right brain with hemiplegia (15 males and 15 females), who were admitted to the Department of Rehabilitation Medicine of Yangpu District Old Hospital, Tianshan Hospital, Shanghai Seventh People’s Hospital and the Neurological Rehabilitation Department of the Hospital of Shanghai University of Sport from February 1, 2012 through March 31, 2014, including 8 cases of cerebral hemorrhage and 22 cases of cerebral infarction.

Inclusion criteria were:

(1) stroke patients with damage of the right half cerebrum must have unilateral limb hemiplegia;
(2) these cases conform to the diagnostic standard of the fourth whole nation cerebrovascular disease academic conference and examinations of CT and MRI of the skull;
(3) be conscious, demonstrating a completely normal ability to listen and understand, stable vital signs within 48 h of first presentation of stroke, and able to actively cooperate with treatment and inspection;
(4) present with first onset of stroke, course of current therapy within 13-month duration;
(5) be right-handed before onset of the disease;
(6) be able to maintain a normal sitting position or posture with normal assistance.

Exclusion criteria were:

(1) no evidence of cerebrovascular disease;
(2) visually impaired;
(3) cases that presented with a consciousness barrier or serious cognitive dysfunction;
(4) not able to maintain a normal sitting position or swing the unaffected forefinger.

#### Patients with Peripheral Nerve Injury

Thirty patients with left upper limb peripheral nerve injury (15 males and 15 females) were selected from cases that were admitted to the Hand Surgery and Rehabilitation Medicine Department of Shanghai Fudan University affiliated Huashan Hospital from December 1, 2012 to August 29, 2014. Cases included eight brachial plexus nerve injury, seven ulnar nerve injury, seven radial nerve injury, and eight median nerve injury.

The diagnostic methods for determining the extent and scope of nerve injury were mainly derived from such variables as the medical history, nerve specialist examination, and electrophysiological examination (Tao and Yang, 2009).

(1) Electromyography (EMG) and nerve conduction function were examined to confirm whether the region of motor units conformed with the neuromuscular action potential (Bendszus et al., 2004), which is considered as the gold standard for evaluating the status of peripheral nerve function (Aagaard et al., 2003);
(2) High-frequency ultrasonography: this technique was applied to diagnose peripheral nerve distribution, course of the condition, breakage level, and defect length;
(3) MRI examination: this test was employed to distinguish the neuraxis rupture and nerve rupture according to the differential signal strength of the STIR (i.e., the nerve and the innervating muscles of T2-weighted images and the short time inversion recovery sequences) and characteristics of the changes at different time points (Yu et al., 2010). Combined with the clinical history details, any patient who met any one of the above three clinical examination evaluation criteria was available for selection and case study.

Exclusion criteria were:

(1) patients that presented with central nerve injury;
(2) upper body does not present with any dysfunction of motor and sensory neurons, reflex action, among other functions;
(3) listening comprehension of the patient is abnormal, and the patient is not able to cooperate effectively with the experimental operation;
(4) the patient was left-handed before the disease.

### Healthy subjects

Sixty healthy subjects that were all right-handed were chosen as a control group from the families of the examined patients.

### Apparatus

**Figure 1:**
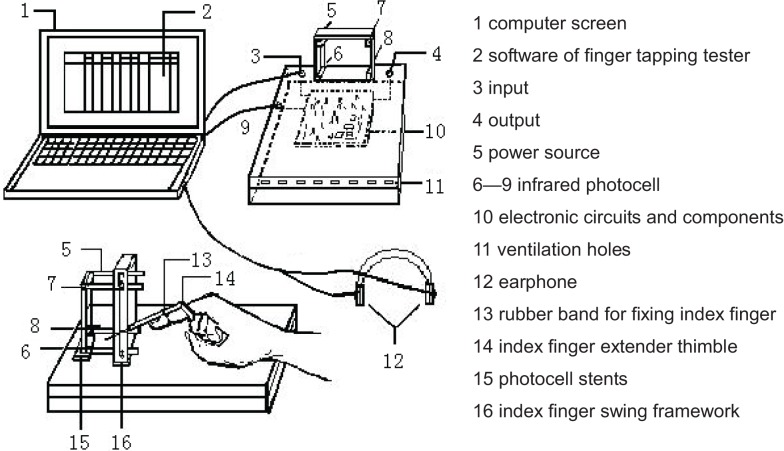
**Sketch of the tester of finger tapping**.

**Figure 2:**
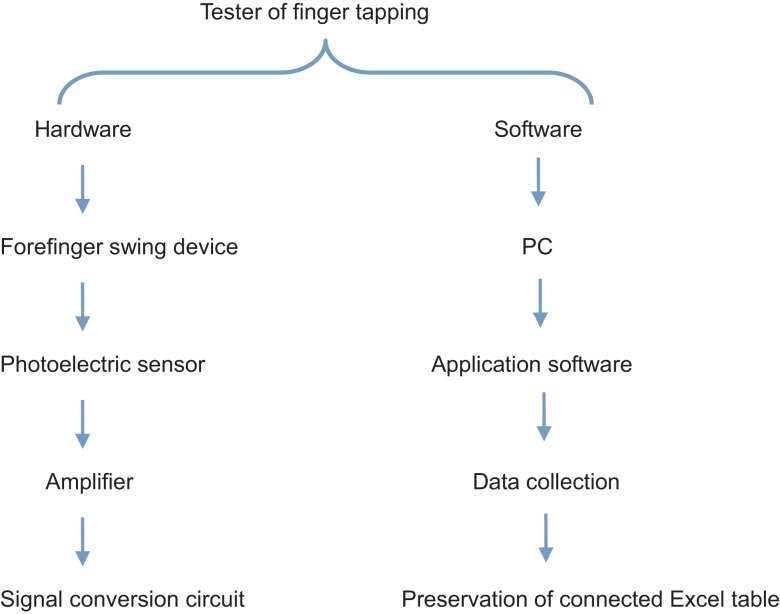
**Representation illustrating the finger-tapping tester**.

The examining technician for finger tapping, which is a method of measuring the speed quality of the human body through the frequency and speed of finger tapping, obtained a patent for the invention, and is an independent intellectual property right of the People’s Republic of China (Figure 1; Yu et al., 2010; invention patent number: 200410017340.1). The theory of applying this approach is the use of the infrared photoelectric sensor to detect finger-tapping movements. The signals obtained will be inputted into the computer via the serial port, with a time accuracy of up to a millisecond (Figure 2).

The test showed that the index finger’s reaction was the fastest, most sensitive, and most flexible link of the human body, and can best indicate the nerve’s incitogram and conduction velocity. The testing technician limits the height of the swing, and controls the angle of the swing up to 30°. The data that do not achieve the height would be identified automatically by the instrument as erroneous data. Excessive swing is restricted by the swing framework. The test time was set to 8 s (10 s or less is the effective time of speed quality in physiological regulation). The finger test can eliminate such factors as muscle strength, speed endurance, and others. Age, gender, major in sports, and result comparisons were contained in the software design, which makes the test results clearer.

### Design and procedure

The testing technicians consulted the medical history following securing the permission from the hospital and selected eligible patients. They made contact with the patients and their families, and explained the purpose, procedure, and relative points of attention. All of the subjects signed the informed consent document. The medical staff explained or ghosted the questionnaire on central nerve injury and the questionnaire on upper limb peripheral nerve injury. We arranged a specific test time after consultation with the subjects, and the subjects could then cooperate to complete the test actively and voluntarily.

Subjects were asked to sit under the normal posture during processing of the test (head straight, eyes staring in front of the swing frame), metacarpophalangeal joint arch, the palm heel and three lateral fingers contacting the desk top, and the index finger extension stretching into the swing frame. To reduce any opportunities for influencing the test results, subjects were asked to put on the unified set of 8 cm long and light extender to adjust the index finger length.

The subjects started a formal test after one to two exercises. The testing technician pressed the start button when the subject was ready. At the sound of the starting pistol, the instrument starts timing automatically and the subject swings his finger rapidly at the same time. In finger-tapping tests, subjects are asked to tap their fingers consistently in a rapid succession (Khan et al., 2014). When the setting time is over, the data are recorded into the computer.

### Data analyses

Participants who completed the finger-tapping frequency test were included in the data analyses. All data were checked for normality using the Shapiro–Wilk test. Descriptive statistics were used to show the characteristics of the participants and their mean age with SD for three groups. The differences of mean finger-tapping frequency among three groups were compared by *post hoc* analysis with Bonferroni-corrected Student’s *t*-tests. Since the finger-tapping frequency was influenced by gender and age, the analysis was separated by gender groups and age was added as a covariate. All of the analyses were conducted using the IBM SPSS Statistics program (formerly SPSS) with version 20.0 software. An alpha *P* value <0.05 was considered statistically significant.

## Results

Age, gender, and group are three major factors that affect finger tapping (Table 1). Finger tapping of the peripheral group’s unaffected hand and the control group’s dominant hand was markedly higher than that of the central group (*P* < 0.05); however, there was no difference between the peripheral group and the control group (Table 2).

**Table 1:** **Comparison of gender and age of the subjects**.

Group	*N*	Sex		Age (years)	Course of disease (months)
		M	F	
Central group	30	15	15	61.10 ± 13.57	3.46 ± 3.04
Peripheral group	30	15	15	43.40 ± 16.69^a^	7.33 ± 5.32
Control group	60	30	30	53.30 ± 8.28^a^,^b^	–

*Data are expressed as mean ± SEM*.

*^a^*P* < 0.05 vs. central group*.

*^b^*P* < 0.05 vs. peripheral group*.

**Table 2:** **Finger-tapping comparison between the central, peripheral, and control groups**.

Group	*N*	Finger tapping
Central group	30	41.30 ± 15.55
Peripheral group	30	59.33 ± 20.61^a^
Control group	60	60.93 ± 10.47^a^

*Data are expressed as mean ± SEM*.

*^a^P < 0.05 vs. the central group*.

By gender, the age from high to low of male subjects was the central group, the control group, the peripheral group, and there were differences among all groups (*P* < 0.05). Finger tapping of the male from high to low was the peripheral group’s unaffected hand, the control group’s dominant hand, and the central group’s unaffected hand, *F*(2,57) = 31.287, Cohen’s *f*  = 0.41. Additionally, finger tapping of the peripheral group’s unaffected hand and the control group’s dominant hand was significantly higher than the central group (*P* < 0.001); however, no difference was seen between the peripheral and control groups (Table 3).

**Table 3:** **Finger-tapping comparison between males and females**.

Sex	Group	*N*	Age (years)	Finger tapping
colorno6M	colorno7Central group	colorno815	colorno960.82 ± 8.54	colorno1039.27 ± 12.47
	colorno11Peripheral group	colorno1215	colorno1335.93 ± 15.43^a^	colorno1470.27 ± 12.35^a^
	colorno15Control group	colorno1630	colorno1754.20 ± 7.46^a^,^b^	colorno1861.07 ± 9.82^a^
colorno19F	colorno20Central group	colorno2115	colorno2261.38 ± 17.61	colorno2343.33 ± 18.35
	colorno24Peripheral group	colorno2515	colorno2650.87 ± 14.82^a^	colorno2748.40 ± 21.71
	colorno28Control group	colorno2930	colorno3052.40 ± 9.07^a^	colorno3160.80 ± 11.26^a^,^b^

*Data are expressed as mean ± SEM*.

*^a^*P* < 0.05 vs. central group*.

*^b^*P* < 0.05 vs. peripheral group*.

The age of the female central group was higher than the peripheral and control groups (*P* < 0.05). Finger tapping of the female from high to low was the control group’s dominant hand, the peripheral group’s unaffected hand and the central group’s unaffected hand, *F*(2,57) = 6.727, Cohen’s *f*  = 0.39. However, finger tapping of the female control group’s dominant hand was significantly higher than the central group’s unaffected hand (*P* < 0.01, *P* = 0.002), the peripheral group’s unaffected hand (*P* < 0.05, *P* = 0.034) (Table 3).

## Discussion

First, our results reveal that the finger-tapping frequency of the unaffected hand of the central group was lower than that of the unaffected hand of the peripheral group and the dominant hand of the healthy group, which illustrated that the healthy hand of the hemiplegic patient and function and fine activities were weakened. It was inferred that the hemi-cerebrum injury might cause a movement disability of the ipsilateral limb. It was reported that there may be some movement controlling disability on the ipsilateral limb when people suffer an attack of stroke. More severely, the disability of the ipsilateral limb is often hidden by the opposite side’s hemiplegia and sensation disorders, which cannot be measured by conventional clinical examination.

By gender, finger tapping by the male or female subject, revealed that the central group’s unaffected hand was significantly lower than that of the control group’s ipsilateral hand. That is because, on the one hand, the ipsilateral cerebral hemisphere has an effect on the ipsilateral limb function, and on the basis of neuroanatomy, 80% of the nerves of one side of the cerebral cortex precentral gyrus cross to the other side and control the opposite limb; while the left uncrossing nerves are situated directly to the anterior corticospinal tract to control the ipsilateral limb.

Therefore, when one side of the cerebral hemisphere is damaged by stroke, the patients’ ipsilateral upper limb and hand function might be influenced because of the existence of the uncrossed nerve fibers (Yin, 2005). The right and left cerebral hemispheres connect through the joint beam. The disordered hemisphere affects the function of the damaged hemisphere, thus affecting the motional control of the contralateral limb, and may also utilize the normal cerebral hemisphere to strengthen and make up for the inadequacy of the function of the affected side. By contrast, it can be seen clinically that the frequency of hemiparalysis normal hand usage decreases in daily life. The family members always would do everything for patients progressively. The excess provision of direct help deprived an opportunity for the patient and desire of participation for daily activities, especially for comparatively more difficult fine motor and coordinated motor functions, which would make the patient rely gradually on others (Dou and Qiu, 1997). All the above observations and inferences would only serve to exacerbate the condition, which is not a good outcome in the context of a functional recovery, and is contrary to the philosophical urban essence of “use it or lose it.”

There also exists differential opinion about whether the function of the hemiplegia patients’ unaffected side decreases. Some physicians and therapists consider that hemiparalysis is a normal limb sensation, and that motion function may decrease to varying degrees. By contrast, others believe that motion function of the hemiplegia patients’ unaffected side will be more flexible because of the increasing use of the normal limb in daily life after damage. The experimental results indicated that unaffected hand function in hemiparalysis is worse than normal ones in healthy at ease, which is consistent with the idea that normal limb sensation of the motion functional responses in “hemiparalysis,” decrease to varying degrees according to Zulin Dou from the Department of Rehabilitation in the Third Affiliated Hospital of Zhongshan Medical University.

Second, our study indicates that there was no significant difference of the finger-tapping frequency between the peripheral patients and the ipsilateral hand of normal healthy controls, which perhaps shows that the peripheral patients’ unaffected fingers do not appear to have had a major impact and can preserve motor function in normal conditions. In the context of gender, finger tapping of the female peripheral group’s unaffected hand was not different from that of the control group’s ipsilateral hand. When considering age, this variable in male peripheral patients was significantly lower than the healthy controls. However, the age of the female between the peripheral patients and the healthy patients was not different. From what has been discussed above, perhaps we might also think that the peripheral patients’ healthy hand function and fine activities have been weakened.

Peripheral nerve is controlled by the central nervous system, but the patients’ contralateral central nerve is not injured and the unaffected hand that is dominated by it has no functional disorder, which suggests that compared with central nerve damage, the segmental nerve damage of the peripheral nervous system has a lesser impact on contralateral limb function. Neural electrophysiological examination and nuclear magnetic resonance examination are mainly used for research on the localization of nerve damage at an early stage, but never for testing the degree of influence of the unaffected limb nerve. Therefore, the impact of the nerve on the motor function of the unaffected limb remains inconclusive. This variability also may result from inter-subject differences in anatomic and physiological factors that affect the independence of the fingers, including biomechanical connections between the digits, functional organization of multitendoned finger muscles, and differences in the central inputs to spinal motor neuron pools (Häger-Ross and Schieber, 2000). Thus, we should increase the number of recruited subjects to determine whether peripheral nerve damage affects the movement function of the contralateral hand.

Third, when people suffer from stroke, they often ignore the normal upper limb’s movement disability of hemiparalysis and rehabilitation, and cannot obtain an accurate and comprehensive assessment and treatment. Therefore, rehabilitation therapists should intensify the practice of the normal upper limb’s fine activities and coordination of the patient. In cases where hemiplegia patients have no hope of obtaining recovery of the affected hand, they should be encouraged to use the contralateral hand for compensation. However, it is noteworthy that it is an error to equate the patients’ “unaffected side” with the healthy people’s “normal side.” That is, our research results show that the emphasis on exercise for the patient’s unaffected side would indirectly affect the rehabilitation of the contralateral side, even by just a minor or marginal affect.

Finally, test evaluation criteria are mainly judged by clinical scales, and the evaluated results may differ considerably because of the medical staff’s subjective assessment factors. This experiment used the testing technician’s finger tapping as the testing instrument and collected finger tapping under the same conditions to decrease experiment error as much as possible, which made the results more scientifically objective and accurate.

## Author Contributions

ZY and LZ designed this study. LZ and PL performed experiments. YL analyzed experimental data. LZ and XH were responsible for manuscript writing. YZ and JZ revised the manuscript. All authors approved the final version of this manuscript.

## Conflict of Interest Statement

The authors declare that the research was conducted in the absence of any commercial or financial relationships that could be construed as a potential conflict of interest.
